# THE CANON - 6

**DOI:** 10.1080/13648470.2012.762339

**Published:** 2013-03-20

Editorial note. Each A&M issue will contain (in no particular sequence) a reappraisal of a past text of what may be considered (unfashionably) canonical, classical or at least of continuing interest in medical anthropology or cultural psychiatry. The sixth is by Horacio Fabrega and Daniel Silver.

**Illness and shamanistic curing in Zinacantan: an ethnomedical analysis**, by Horacio Fabrega, Jr. and Daniel B. Silver, Stanford, California, Stanford University Press, 1973

Piggy-backed onto the existing Harvard University Chiapas Project in South-Western Mexico, Fabrega and Silver initially attempted a rather wider survey in Zinacantan using sociological and psychological theory, but were forced to restrict themselves to looking at just one occupational group – the *h'iloletik* or shamans. They continued to use quantitative data recorded in many tables and projective psychological tests, and the book may be seen as on the cusp between the earlier American Culture and Personality School and the newer cognitive and medical anthropologies that emerged from that position.

They start by contrasting two ways of looking at medical issues in ‘isolated and non-literate groups’: (i) the first is the epidemiological approach to biomedical disease (and a derivative of this – the study of local biologies); (ii) and what they term the ethnomedical, an examination of local subjectivities and recourse to healing, which they argue is properly part of anthropology. They attempt to follow the latter but recognise that this is still to be anchored in biomedical frames of reference rather than, say, in local cosmology; this seems to be in part as a consequence of a rather particularised method of individual specific studies such as population surveys, questionnaires and the psychological tests (p. 12). (Whether this is their choice or else part of the constants of the Harvard Chiapas Project we do not learn, but they do not stray very far from the biomedical starting point, constantly urging that biomedical disease has to be the departure point.) They warn against the assumptive that the cultural variables the anthropologist is interested in will themselves all stem from ‘culture’, and note that the effective healer may well be familiar with the signs of good or bad prognosis in the biological domain – for their efficacy will be greater when dealing with the former. Following Frank and Kiev they argue that healing is ‘persuasion’ in a heightened emotional setting.

They survey the earlier assumption that healers such as shamans are conspicuously deviant or have an ‘underlying psychotic personality … somehow protected and concealed by the behavioural requirements of their role’ (see Canon – 2). The *h'iloletik* comprise 40 of the local population (predominantly male) who are called in dreams by the local Maya gods, who exist with Christianity. The signs of election are often seizures, and a period of covert practice is followed by public recognition that involves onerous ceremonial duties. Shamans perform a wide role, which is contrasted with the local midwives, bone setters and owners of ‘talking saints’ (which are divinatory religious pictures). The authors back up these points with lots of Chi-square tests and tables: for instance on shamans having shamans disproportionately among their relatives. Their extensive data seem largely derived from formal surveys rather than interaction with the locals. There are few narrative stories here. A detailed questionnaire comparison includes Ink Blot projective tests, which show little difference between shamans and non-shamans except in the areas of ‘emotional loading’ and perception of human figures in the blots. Fabrega and Silver conclude statistically that shamans are not especially abnormal compared with non-shamans in the community.

With the exception of a Hot/Cold dichotomy, which runs through the body, and through sickness and treatment, there are few ideas of naturalistic aetiology in shamanic thinking, nor a distinction between material and ‘psychological’ illness. Bush remedies are prescribed for conditions because of their ‘supernatural’ significance. There is a slippage between different causal models in an illness (e.g. between that sent directly by a witch mediated by a witch and that sent by the gods) and hence little specific treatment. There is little local sense of an autonomous self, and emotions are seen as purely circumstantial. (This is of interest given Fabrega's later and influential arguments in favour of an autonomous Cartesian self as significant in schizophrenia.) Starting from existing studies on ethnoscience by Frake, Pike, Conklin and Berlin, the authors extend the already established distinction between a ‘disease’ and an ‘illness’, although they are still closer to the former for comparative purposes: ‘… refers to the common cold’, ‘may be equivalent to malaria’, ‘can be translated as …’. Admittedly other local categories are identified solely through the local description. ‘Accuracy’ of diagnosis is measured by getting Zinacantan ecos to diagnose disease by assessing medical photographs. Taking all previously recorded illness categories, they correlate them with the elicited symptoms and find a fair (but not universal) fit with human pathophysiology. Coefficients demonstrate relatively little consistency between individuals on the clustering of particular symptoms as categories.

A more descriptive method is used to portray the major healing ceremonies, which use candles, herbal baths, flowers gathered on various local pilgrimage points, such as mountains and churches, and which are used to return sorcery onto its unknown sender.

Fabrega and Silver do not provide a ‘full’ social ethnography, presumably because of the coexistent Harvard project to which they constantly refer. Their conclusions? That healing mediates disputes and resolves conflicts (but they give no examples). Participants at the ceremonies beg pardon for transgressions and apologise to each other (similar to Victor Turner's Ndembu healing, which he published a few years previously). The healer knows the local community well (again, as Turner) and that most illnesses are attributed to witchcraft or the patient's transgressions (both of which they argue signal social disturbance). The proximate cause is a failure to propitiate the Gods (a failure of community obligations), which is diagnosed by the shaman who mediates between human and ultra-human worlds. The authors refrain from offering a simple functionalist analysis but point out that healing ceremonies distribute surplus wealth, which had often been the reason for the failure to observe community obligations through the ceremonial *cargo* responsibilities. Recourse to the shaman, who is respected but feared, is less arduous and controversial than appealing to the courts or police.

They end by criticising Ackernecht's earlier emphasis on distinguishing local explanations from the rationality of Western biomedicine, pointing out (like Evans-Pritchard) that local explanations are quite ‘rational’ given different epistemological premises. They affirm that a language for medical anthropology, based on local responses to and considerations of biological variables, can be derived from studies elsewhere, and once refined can lead to successive comparative analysis. This, of course, has not happened, as we seem to have moved to a more ‘social concerns located in the body’ position. But Fabrega and Silver's interest in psychology and individual motivation seems to have appeared in the recent (and particularly North American) interest in subjectivity. And a concern with local biologies has also emerged, even if our two authors once bracketed this with epidemiology.

Roland Littlewood

University College London, UK

*Email:* r.littlewood@ucl.ac.uk

© 2013, Roland Littlewood

